# Neutron flux evaluation model provided in the accelerator-based boron neutron capture therapy system employing a solid-state lithium target

**DOI:** 10.1038/s41598-021-87627-8

**Published:** 2021-04-13

**Authors:** Satoshi Nakamura, Hiroshi Igaki, Masashi Ito, Shoji Imamichi, Tairo Kashihara, Hiroyuki Okamoto, Shie Nishioka, Kotaro Iijima, Takahito Chiba, Hiroki Nakayama, Mihiro Takemori, Yoshihisa Abe, Tomoya Kaneda, Kana Takahashi, Koji Inaba, Kae Okuma, Naoya Murakami, Yuko Nakayama, Mitsuko Masutani, Teiji Nishio, Jun Itami

**Affiliations:** 1 Department of Medical Physics, National Cancer Center Hospital, Tsukiji 5-1-1, Chuo-ku, Tokyo, 104-0045 Japan; 2 Division of Research and Development for Boron Neutron Capture Therapy, National Cancer Center Exploratory Oncology Research & Clinical Trial Center, Tsukiji 5-1-1, Chuo-ku, Tokyo, 104-0045 Japan; 3 Department of Radiation Oncology, National Cancer Center Hospital, Tsukiji 5-1-1, Chuo-ku, Tokyo, 104-0045 Japan; 4 Department of Radiology, National Center for Global Health and Medicine, Toyama 1-21-1, Shinjuku-ku, Tokyo, 162-8655 Japan; 5 Division of Cellular Signaling, National Cancer Center Research Institute, Tsukiji 5-1-1, Chuo-ku, Tokyo, Japan; 6 Department of Radiological Science, Graduate School of Human Health Sciences, Higashi-ogu 7-2-10, Arakawa-ku, Tokyo, 116-8551 Japan; 7 Department of Radiological Technology, National Cancer Center Hospital, Tsukiji 5-1-1, Chuo-ku, Tokyo, 104-0045 Japan; 8 Department of Molecular and Genomic Biomedicine, Nagasaki University Graduate School of Biomedical Sciences, Sakamoto 1-7-1, Nagasaki, 852-8588 Japan; 9 Division of Health Science, Graduate School of Medicine, Osaka University, Yamadaoka 1-7, Suita-shi, Osaka, 565-0871 Japan

**Keywords:** Medical research, Physics

## Abstract

An accelerator-based boron neutron capture therapy (BNCT) system employing a solid-state Li target can achieve sufficient neutron flux for treatment although the neutron flux is reduced over the lifetime of its target. In this study, the reduction was examined in the five targets, and a model was then established to represent the neutron flux. In each target, a reduction in neutron flux was observed based on the integrated proton charge on the target, and its reduction reached 28% after the integrated proton charge of 2.52 × 10^6^ mC was delivered to the target in the system. The calculated neutron flux acquired by the model was compared to the measured neutron flux based on an integrated proton charge, and the mean discrepancies were less than 0.1% in all the targets investigated. These discrepancies were comparable among the five targets examined. Thus, the reduction of the neutron flux can be represented by the model. Additionally, by adequately revising the model, it may be applicable to other BNCT systems employing a Li target, thus furthering research in this direction. Therefore, the established model will play an important role in the accelerator-based BNCT system with a solid-state Li target in controlling neutron delivery and understanding the neutron output characteristics.

## Introduction

The clinical outcomes after treating patients with boron neutron capture therapy (BNCT) have been reported in various studies^1–10^, and the biological effectiveness of killing cells via BNCT has also been demonstrated in biological in vivo and in vitro experiments^11–16^. In BNCT, the treatment efficacy is based on the ^10^B(n, α)^7^Li reactions. Thus, neutron irradiation to the patient is performed after compounds containing ^10^B, such as boronophenylalanine (BPA) and sodium borocaptate (BSH), are administered^7–9^. Because the ^10^B concentration in the tumor cells is expected to be a few times higher than that of the surrounding normal tissues, the tumor cells are selectively damaged by the generated particles, whose ranges are comparable to the cell size. Because the nuclear daughter products (^4^He and ^7^Li) exhibit high linear energy transfer, the relative biological effectiveness of BNCT is greater than that of conventional radiotherapies that use photons and electrons^17^. Thus, BNCT is expected to effectively kill cells that are resistant to conventional radiotherapies when the boron compounds have sufficiently accumulated in the cells^13,15^. Using these properties, several clinical studies have been conducted in nuclear reactors, such as Kyoto University^1–4,18,19^, and remarkable clinical outcome of BNCT has been demonstrated in those studies^1–10^. However, nuclear-reactor-based BNCT is not employed worldwide owing to the difficulty of using nuclear reactors as neutron sources for BNCT.

Recent studies have indicated that accelerator-based neutron sources can achieve sufficient neutron flux for use in BNCT^20–24^. Two main types of accelerator-based neutron sources are generally considered to acquire neutrons for accelerator-based BNCT systems. To generate the neutrons, one source utilizes the reaction of ^7^Li(p, n)^7^Be, whereas the other source utilizes the reaction of ^9^Be(p, n)^9^B^20–26^. In the former system, the maximum generated neutron energy is below 1 meV and the incident proton energy of around 2.5 meV is generally considered, whereas that for the latter system is higher by a few MeV (i.e., the incident proton energy is higher than 8 meV)^20–24^. Therefore, the advantage of the former system is that the lower neutron energy facilitates a more compact BNCT system as the generated neutrons can be easily moderated to produce the ideal neutron energy (approximately 10 keV). This is related to reduced shielding requirements and moderator for the system. However, a disadvantage is that the melting point of Li is lower than that of Be as a high thermal loading is required on the Li target^20,27,28^. Considering these properties, the National Cancer Center Hospital (NCCH), Tokyo, Japan, is conducting a clinical study using an accelerator-based BNCT system employing the Li target to evaluate its efficacy in clinical oncology^20,23,24^.

In an accelerator-based BNCT system employing a Li target, a huge number of the ^7^Li(p, n)^7^Be reaction is required to acquire a sufficient number of neutrons, which can result in a high thermal loading^20,27,28^. Previous studies suggested that the degradation of the Li target, such as thinning and damage, could be expected owing to ion-impacts, high operating temperatures, and other factors resulting from proton bombardment. In this study, the thinning of the Li target was defined as the shorten proton interaction path length in the Li target. This could then induce a reduction in the neutron production efficiency per unit of proton current^20,27,28^. Hence, it is important for the accelerator-based BNCT system employing the Li target in which the neutron output characteristics are evaluated to control for the neutron fluence to the patient. This study aims to investigate the neutron output characteristics and establish a model for the neutron flux using an accelerator-based BNCT system employing a solid-state Li target. Introduction section is followed by the methods, wherein different approaches for the measurements/calculations/theoretical models are described. This section is followed by the results and discussions part, where the outcomes of measurements/calculations/theoretical models are presented with elaborated discussions. The article is summed up in the conclusions section with significant outcomes and future scope/limitations.

## Methods

The experiments were performed using an accelerator-based BNCT system (Cancer Intelligence Care Systems (CICS), Inc., Tokyo, Japan) at NCCH, which employs a solid-state Li target made of natural lithium metal. The system consists of an accelerator for the proton, transport devices for the accelerated protons, a target sample (containing the Li target), and beam shaping assembly (BSA). A detailed description of the system has been reported in a previous article^20^. A nominal proton current of 12 mA is delivered to the target sample, and its nominal energy is 2.5 meV^24^. The neutrons are then generated by the reaction between the Li target and the delivered protons. Hence, the maximum energy of the generated neutrons is 786 keV at 0 degrees in the lab^20,23–26^ because the *Q*-value of the ^7^Li(p, n)^7^Be reaction is − 1.64 meV^25,29^. After the generated neutrons pass through the BSA that is made of MgF_2_ (Nippon Light Metal Co. Ltd., Tokyo, Japan, and CICS)^30^, the generated neutron energy is moderated to an ideal energy, of 10 keV for BNCT.

**Figure 1:**
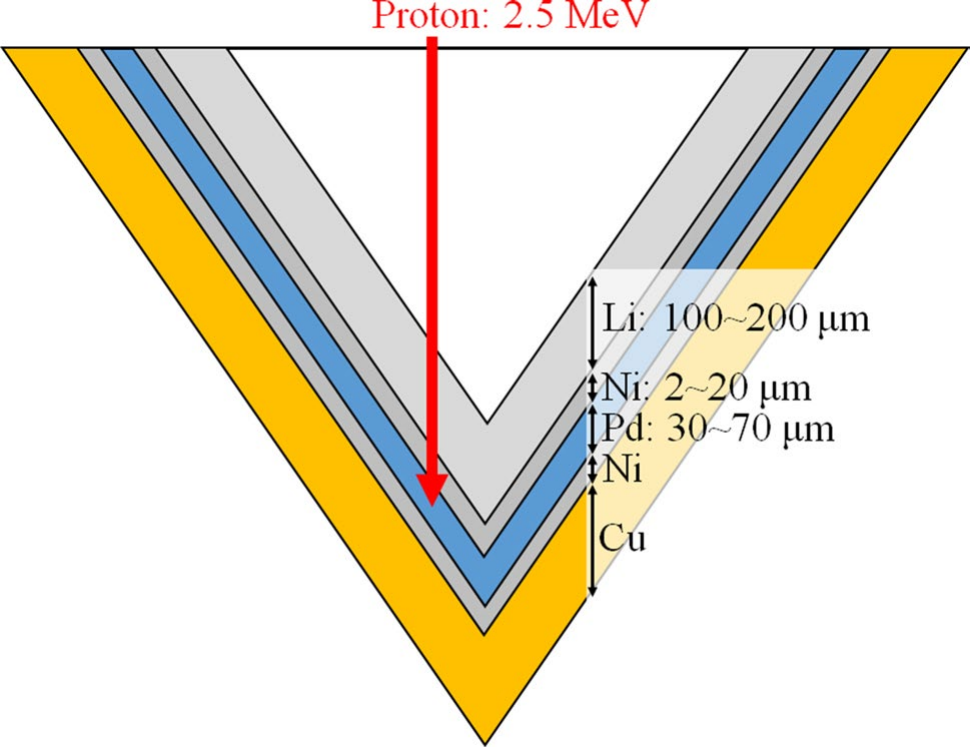
A schema of a cross-sectional view of the target sample.

The target sample consists of the Li target, first Ni layer, Pd layer, second Ni layer, and a copper support. A schema of the cross-sectional view of the target sample is presented in Fig. 1.

The nominal thickness of the Li target along the incident proton angle reaches more than approximately 100 μm^20^. In our previous report, the Li thickness greater than 90 μm did not significantly affect the neutron production efficiency per unit of proton current^23^. This is because a Li thickness of 90 μm is required to decelerate the incident proton with an energy of 2.50 meV to the threshold energy of the ^7^Li(p, n)^7^Be reaction. If the proton beam passes through the Li thickness greater than 90 μm, then the residual proton energy is below the threshold energy and does not affect the neutron production. In the target sample, a large thermal load is expected and effective methods for removing it are adopted. The detailed description of the target sample has been reported in our previous article^20^.

The accelerator-based BNCT system at NCCH generates neutrons via the reactions of ^7^Li(p,n)^7^Be. Hence, the number of generated neutrons depends on the number of protons delivered to the target sample. Thus, using the five target samples, the neutron flux was evaluated per unit of proton current in this study^20,23^. Each of the five target samples had the geometry and parameters as given in Fig. 1 and the manufacturing process was same. To each target an individual lot number was given and the target behavior and found results, such as the degree of degradation and the neutron production efficiency per unit of proton current, were related to this different lot numbers.

### Measurement of neutron flux per unit of proton current

According our previous study, the total neutron flux depended on the variation of Li thickness^23^. Based on the simulated spectrum of neutrons, the cross-section for the reaction of ^197^Au(n, g)^198^Au, and the measurement result, the total neutron flux for each Li thickness was correlated with the corresponding saturated radioactivity in the accelerator-based BNCT system employing the Li target^23^. Hence, the saturated radioactivity of gold could be a substitute for the total neutron flux in the accelerator-based BNCT system even if the degradation of the Li target was induced^23^. Therefore, the saturated radioactivity of gold was measured to evaluate the neutron flux in this study. A gold wire with a 0.5-mm diameter and 8.0-mm length was utilized in this study. The purity of the gold wire is 99.5% of natural gold. The gold wire was placed at the center of the bottom surface of the irradiation port of the system, and the neutrons were then delivered. The experimental geometry is consistent with our previous report^20^. To investigate the relationship between the number of generated neutrons and the number of protons delivered to the target sample, a relationship was used to evaluate the saturated radioactivity of the metal in units of Bq/mA/atom. This relationship was evaluated as Eq. (1).

1 $$SA=\frac{Activity}{N\times \sum_{i=1}^{n}{A}_{i}\times \left(1-{\mathrm{e}}^{-\lambda \times T}\right)\times {\mathrm{e}}^{-\lambda \times \left(n-i\right)\times T}} \quad [\mathrm{Bq}/\mathrm{mA}/\mathrm{atom}],$$

where $SA$ denotes the saturated radioactivity of the metal, $Activity$ denotes the induced radioactivity of the metal at the end of the neutron irradiation to the metal, $N$ denotes the number of atoms in the metal, $A$ denotes the proton current, $\lambda$ denotes the decay constant of the induced radioactivity, and $T$ denotes one of the neutron irradiation time, which have been divided into n. In this study, $T$ is determined as 1 ms. This evaluation can eliminate the effects of the fluctuations of the delivered protons, the individual difference in each gold wire, and the radioactive decay in each measurement.

During the neutron exposure to the gold wire, the number of protons delivered to the target sample was measured using two ammeters (NPCT-CF6, Bergoz Instrumentation, Saint-Genis-Pouilly, France) equipped with a transport device. To measure the induced radioactivity, the proton charge of 3.6 × 10^3^ (range: (3.2–4.2) × 10^3^) mC were delivered to the target sample. It was equaled to 5 min irradiation. The reaction between the gold wire and neutrons induced the formation of ^198^Au that subsequently emitted 412 keV gamma rays per decays. In this study, the number of emitted gamma rays was measured using a high-purity germanium (HP-Ge) detector, and the number of induced radioactivity (^198^Au) within the gold wire was then determined. The detection efficiency of the HP-Ge detector for the induced ^198^Au was calculated via Monte Carlo simulation (GEANT4, ver. 10.1)^31–33^. The measurement geometry using the HP-Ge detector was reproduced from the simulation to calculate the detection efficiency. The HP-Ge detector was already modeled using radioactive sources that were validated by the Japan Radioisotope Association, Tokyo, Japan. This has already been reported in a previous study^34^. The events resulting in the reactions between the induced radioactivity and the HP-Ge detector were counted using a multichannel analyzer (MCA7600, SEIKO EG&G, Tokyo, Japan), and Gamma-studio software (SEIKO EG&G, Tokyo, Japan) was used to calculate the number of photoelectric events from all of the events that occurred in the HP-Ge detector.

According to previous studies, the neutron flux was reduced depending on the total number of protons delivered to the target sample^17,20^. Previous studies also suggested that this is as a result of gradual thinning of the Li thickness caused by the thermal load on the target sample^20,28^. Thus, this study evaluated the neutron flux based on the total number of protons delivered to the target sample. The integrated proton charge of 2.70 × 10^6^ mC was delivered on the target sample to examine the neutron flux because a lifetime of each Li target was 2.52 × 10^6^ mC in the system and the safety margin of 1.80 × 10^5^ mC was considered. In each evaluation interval, the accumulated proton charges that were delivered to the target sample were not greater than 86.4 × 10^3^ mC, which was equaled to 2 h irradiation.

### Establishing a model for the neutron flux using the relationship between the number of generated neutrons and the total number of protons delivered to the target sample

Lee and Zhou reported a method for calculating the total neutron yield from the Li target that had sufficient thickness to generate neutrons, but not enough to decelerate the residual proton energy below the reaction threshold energy after passing through Li’s thickness^25^. If an accelerated proton stops in the Li target, it may result in blistering, and damage of the Li target^35^. For this reason, a thin Li layer is adopted for the Li target to allow accelerated protons to pass through the Li target. In this study, the total neutron yield for each thickness of the Li target was calculated using Lee and Zhou’s method^25^. The method was as follows:

2 $${Y}_{\mathrm{partial}}=\frac{{f}_{{7}_{\mathrm{Li}}}{N}_{0}}{e{A}_{\mathrm{eff}}}{\int }_{{E}_{\mathrm{p},\mathrm{ exit}}}^{{E}_{{\mathrm{p}}_{0}}}\frac{{\sigma }_{\mathrm{pn}}({E}_{\mathrm{p}})}{-\frac{1}{\rho }\frac{d{E}_{p}}{dx}}d{E}_{\mathrm{p}} \quad [\mathrm{mC}^{-1}],$$

where *Y*_partial_ represents the total neutron yield from a partially thick target, ${f}_{{7}_{\mathrm{Li}}}$ represents the ^7^Li atomic fraction in the natural lithium metal, *N*_0_ represents Avogadro’s number, *e* represents the electronic charge, *A*_eff_ represents the atomic weight of the natural lithium metal, ${E}_{{\mathrm{p}}_{0}}$ represents the incident proton energy, *E*_p, exit_ represents the residual proton energy after passing through the Li target, *σ*_pn_(*E*_p_) represents the total cross-section of the ^7^Li(p,n)^7^Be reaction at each of the residual proton energy (*E*_p_), *ρ* represents the physical density of the Li target, and *dE*_p_*/dx* represents the mass-stopping power of protons in the Li target at each of the residual proton energy (*E*_p_)^25^. In this study, ${f}_{{7}_{\mathrm{Li}}}$, *N*_0_, *e*, *A*_eff_, ${E}_{{\mathrm{p}}_{0}}$, and *ρ* were set at 0.925, 6.022 × 10^23^ mol^–1^, 1.602 × 10^–19^ C, 6.941 u, 2.50 meV, and 0.534 g/cm^3^, respectively. The total cross-sections of ^7^Li(p, n)^7^Be reaction at each of the proton energies were derived from the report from Liskien and Paulsen (E_p_ ≥ 1.93 meV)^29^ and Lee and Zhou (E_p_ < 1.93 meV)^25^. The values of *dE*_p_*/dx* were calculated via Monte Carlo simulations (using SRIM-2013^36^) at proton energies ranging from 1.88 to 2.50 meV, and the gaps in the total cross-section and *dE*_*p*_*/dx* at specific energies were interpolated. In the simulations, a virtual Li thickness greater than 250 μm was adopted and it was sufficiently thick to evaluate the Bragg peak in order to validate the calculations. Hence, the virtual Li thickness did not correspond to the Li target. The total cross-section and *dE*_p_*/dx* at each proton energy were calculated in 10.0 keV intervals in the residual proton energy range from 1.88 to 2.50 meV, as the proton energy of 1.88 meV was reported as the threshold energy of ^7^Li(p, n)^7^Be reaction^25,29^. Therefore, the total neutron yields for various residual proton energies after passing through the Li target can be calculated. Additionally, the reaction of ^7^Li(p, n)^7^Be has a resonance cross-section at an incident proton energy of 2.25 meV^29^. The energy width of the resonance is 2.15 to 2.50 meV^29^. Thus, in this study, the total neutron yield was evaluated in two residual proton energy regions. The first one corresponded to the residual proton energy between 2.15 and 2.50 meV, and it considered the total neutron yield derived from the resonance reaction. The other one corresponded to the residual proton energy between 1.88 and 2.15 meV, and it considered the total neutron yield derived from both the resonance reaction and the reaction ranging from the threshold to resonance energy.

In the study by Lee and Zhou, the total neutron yield (*Y*_partial_, Eq. (2)) was computed as a function of the proton energy before and after passing through the Li target. In the accelerator-based BNCT system employing the Li target, an incident proton energy of 2.5 meV is generally considered, and is also applied in this study^20,23,24,35,37–39^. This is because the neutron generation reaction of ^7^Li(p,n)^7^Be* must be considered when an incident proton energy of more than 2.5 meV is adopted^29^. Hence, the total neutron yield can be computed as a function of the residual proton energy after passing through the Li target. However, the residual proton energy was not convenient for investigating the neutron output characteristics in the accelerator-based BNCT system because the neutron flux was reduced owing to the thinning of the Li thickness. Thus, this study investigated the total neutron yield as a function of the Li target thickness. The energy depositions and the residual proton energies after passing through a certain Li thickness were calculated via Monte Carlo simulations (using SRIM-2013). Therefore, *Y*_partial_ (Eq. (2)) was modified as a function of the Li target thickness (${Y}_{\mathrm{partial}}^{\mathrm{^{\prime}}}$).

3 $${Y}_{\mathrm{partial}}^{\mathrm{^{\prime}}}\left(t\right)=\frac{{f}_{{7}_{\mathrm{Li}}}{N}_{0}}{e{A}_{\mathrm{eff}}}{\int }_{{t}_{\mathrm{p},\mathrm{ exit}}}^{0}\frac{{\sigma }_{\mathrm{pn}}\left({t}_{\mathrm{p}}\right)}{-\frac{1}{\rho }\frac{d{E}_{\mathrm{p}}\left({t}_{\mathrm{p}}\right)}{dx}}d{t}_{\mathrm{p}} \quad [\mathrm{mC}^{-1}],$$

where ${Y}_{\mathrm{partial}}^{\mathrm{^{\prime}}}$(*t*) denotes the total neutron yield in the Li target thickness of *t* μm, ${t}_{\mathrm{p},\mathrm{ exit}}$ denotes the Li target thickness, ${\sigma }_{\mathrm{pn}}\left({t}_{\mathrm{p}}\right)$ denotes the total cross-section of the ^7^Li(p, n)^7^Be reaction at the residual proton energy after passing through a certain Li thickness (*t*_p_), and *dE*_p_(*t*_p_)*/dx* denotes the mass-stopping power of the residual proton energy after passing through a certain Li thickness (*t*_p_).

It is not convenient to utilize Eq. (3) for the model to determine the neutron output characteristics because there are numerous variables. Therefore, Eq. (3) was simplified in this study. Additionally, previous reports have suggested that the reduction of the neutron flux per unit of proton current is caused by protons delivered to the target sample, and it may then induce the degradation of the Li target^20,27,28^. The degradation was proportional to the thermal load owing to the proton bombardment^27^. Hence, in this study, the thermal loads on the Li target were quantified based on the total energy depositions, and the variation of Li thickness was then considered as a reason for the reduction of the neutron flux. Considering the number of protons delivered to the target sample (proton current of 12.0 mA in this study), the total energy deposition in each Li thickness was then evaluated. The total energy depositions after passing through each of the Li thicknesses were calculated through the Monte Carlo simulation (SRIM pro 2013). To simplify the model for the neutron output characteristics, the thickness of the Li target was utilized instead of the number of Li atoms in the Li target, and the total neutron yield as a function of the number of protons delivered to the target sample was then computed. Therefore, a model for the neutron flux was established. Note that the cooling efficiency of the Li target has not yet been considered and is left for a subsequent study.

### Validation of the model for neutron flux using the measured neutron flux

The model for the neutron flux was applied to the measured neutron flux per unit of proton current based on the total number of delivered protons in each of the five target samples. Using the least-squares method, several coefficients in the model were determined. Hence, the calculated neutron flux at a certain total number of protons delivered to the Li target could then be evaluated by the model.

The calculated neutron flux was compared with the measured neutron flux along the total number of protons delivered to the target sample. These results were then computed to examine whether the neutron output characteristics could be expressed by the model. A Shapiro–Wilk test was performed to examine whether the discrepancies between the measured and calculated neutron flux followed a normal distribution. Additionally, the discrepancies among the five target samples were compared, and Bartlett’s test was used to examine whether the discrepancies followed a normal distribution. A one-way analysis of variance (ANOVA) as a parametric test and a Kruskal–Wallis test as a non-parametric test were applied to these results.

This study also examined that the model was derived from the measured neutron flux at the integrated proton charge of up to 60, 100, 200, 300, 400, 500, and 600 mA × h, respectively, and the neutron flux after the amount of delivered protons of 12 mA × h (i.e., 12 mA × 1 h irradiation) was then calculated. It was compared with that was calculated via the model derived from the measured neutron flux at the integrated proton charge of up to 750 mA × h.

In this study, cross-validation for the neutron flux model was also performed using the five target samples whether the individual difference among the five targets affected the neutron production. The neutron flux model for the cross-validation was established by the measured neutron flux in four target samples, and its neutron flux model and the neutron flux measured in the fifth target sample were then compared based on the total number of delivered protons. These results were then computed to examine whether the neutron output characteristics could be expressed by the model established by the other four target samples. In each of the five target samples, its method for cross-validation was applied. This was performed to determine whether the coefficients in the model should be established by each target sample. A Shapiro–Wilk test was performed to examine whether the discrepancies between the measured and calculated neutron flux, using the neutron flux model established by the other four target samples, followed a normal distribution. Additionally, the discrepancies among the five target samples were compared, and Bartlett’s test was used to examine whether the discrepancies followed a normal distribution. A one-way ANOVA as a parametric test and a Kruskal–Wallis test as a non-parametric test were applied to these results. A *P*-value of less than 0.05 was considered statistically significant in this study.

## Results

### Measurement of neutron flux per unit of proton current

**Figure 2:**
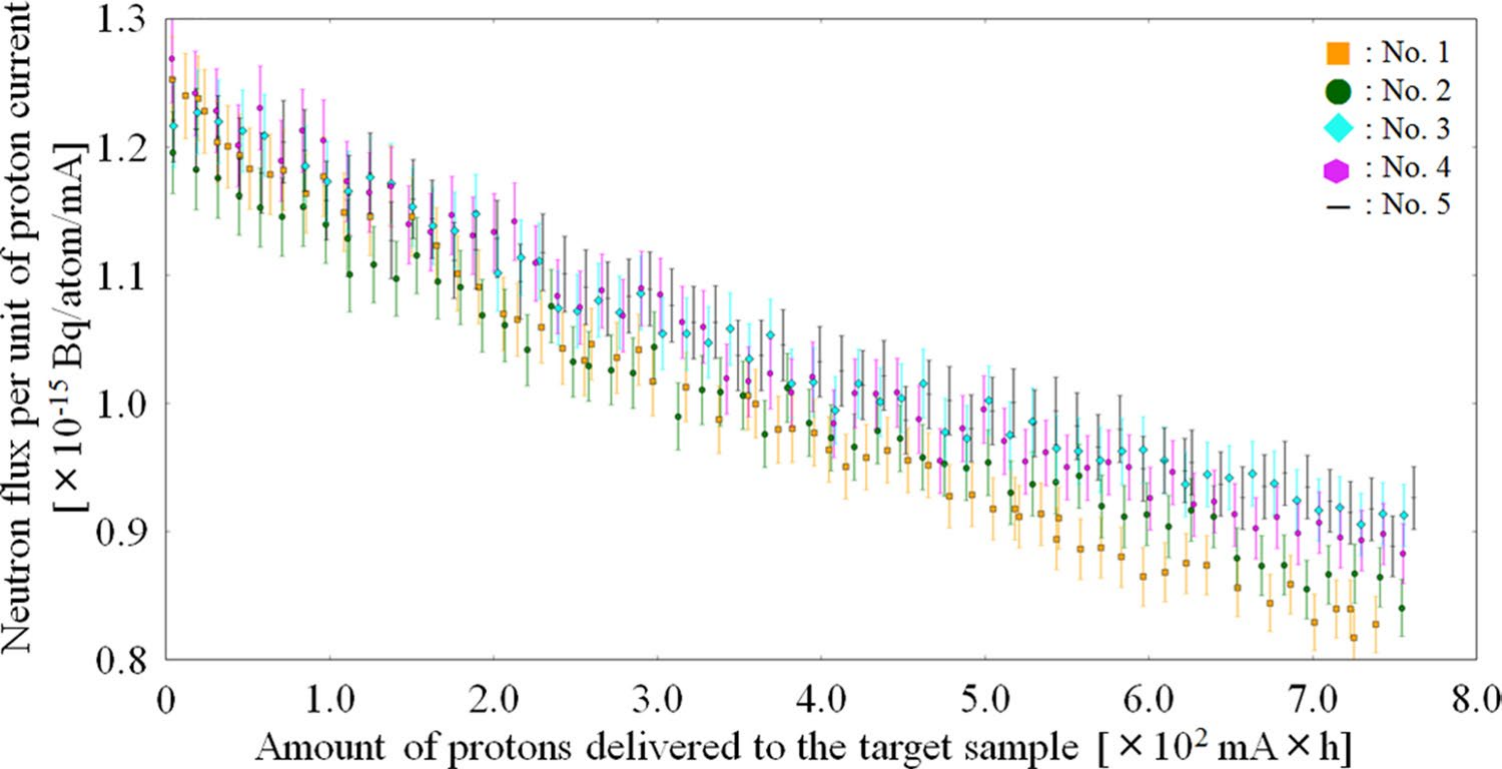
Measured neutron flux per unit of proton current in each target sample. The saturated radioactivity of ^198^Au was used as a substitute for the neutron flux.

Figure 2 shows the measured neutron flux per unit of proton current against the total number of delivered protons in each target sample.

According to Fig. 2, the measured neutron flux in each target sample reduced as a function of the total number of protons delivered to the target sample. The reduction reached 28% (range: 24–32%) in each target sample when the integrated proton charge of 2.52 × 10^6^ mC was delivered. It was equaled to 58.3 h irradiation.

### Establishing a model for the neutron flux using the relationship between the number of generated neutrons and the total number of protons delivered to the target sample

**Figure 3:**
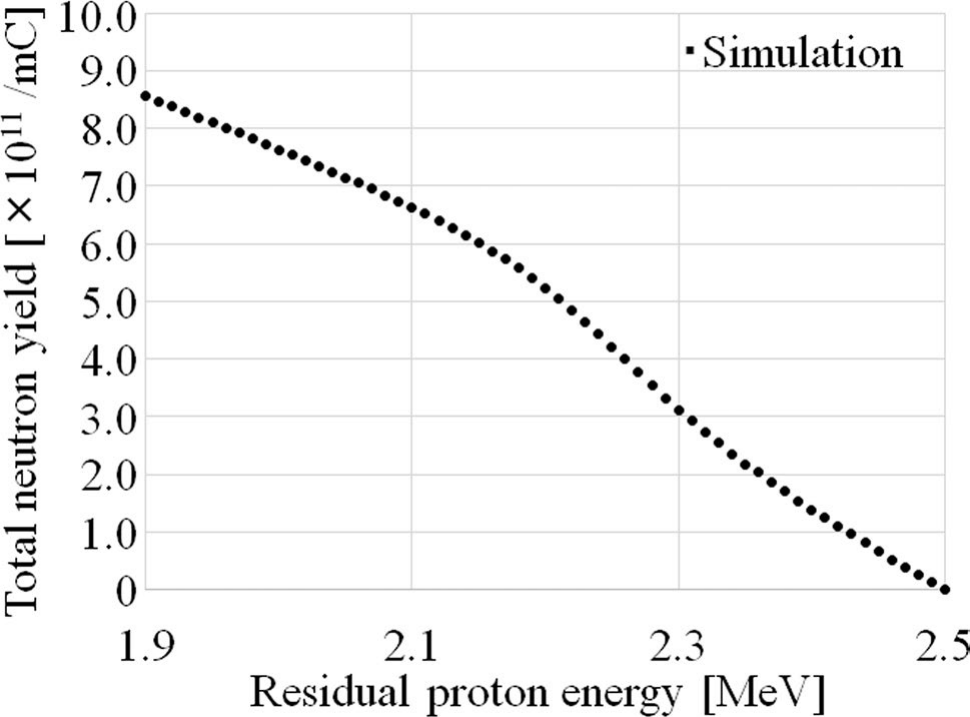
Total neutron yield as a function of the residual proton energy after passing through the Li target. The incident proton energy was 2.5 meV.

The total neutron yield as a function of the residual proton energy after passing through the Li target is shown in Fig. 3. In the residual proton energy ranging from 1.88 to 2.15 meV, the total neutron yield was approximately proportional to *E*_p, exit_ (i.e., the total neutron yield decreased linearly with increasing *E*_p, exit_).

**Figure 4:**
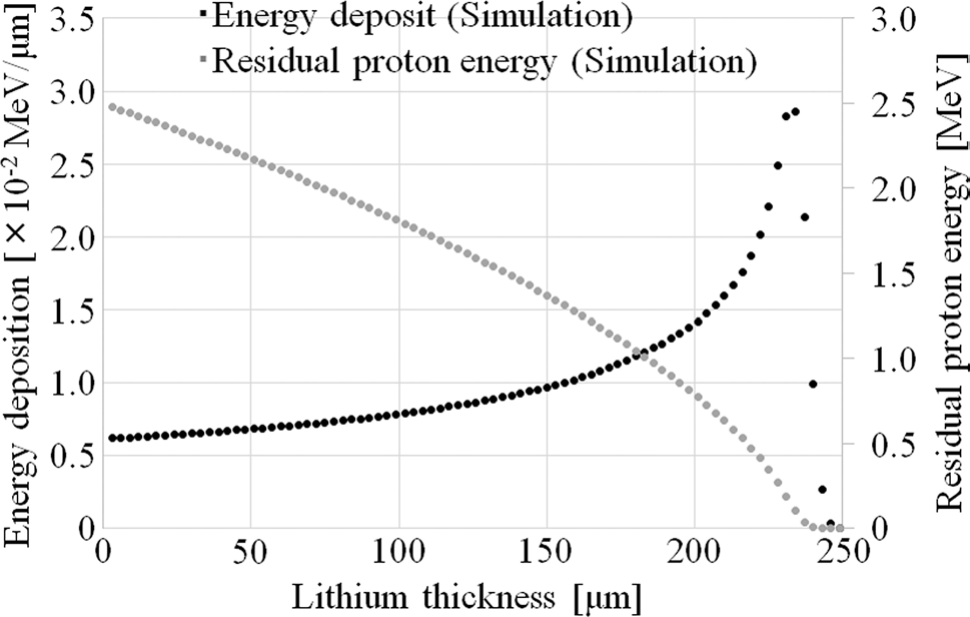
Proton energy depositions and residual proton energies at each Li thickness. The incident proton energy was 2.5 meV.

Figure 4 shows the proton energy depositions and the proton residual energies in the Li target against the Li thickness. A Bragg peak was observed at the lithium thickness of approximately 230 μm.

**Figure 5:**
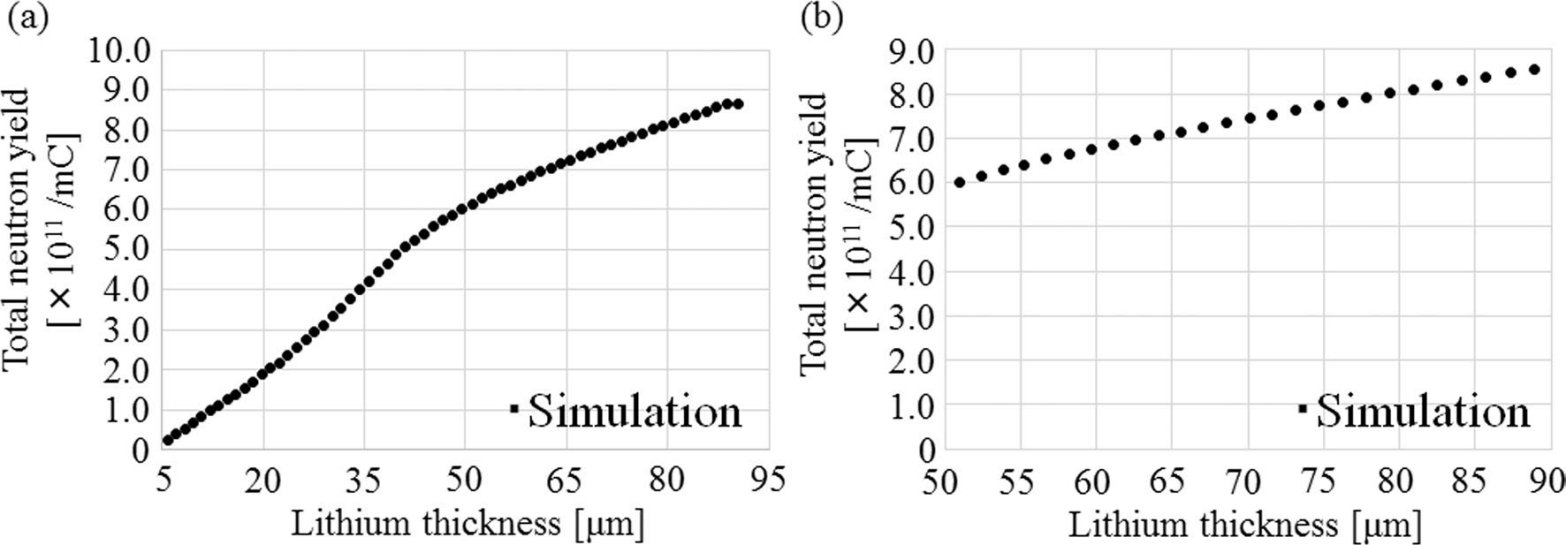
(**a**) Total neutron yield as a function of Li thickness on the Li target. (**b**) Total neutron yield versus the Li thickness in the range from 50 to 90 μm.

Considering the Fig. 3 and 4, the total neutron yield can be calculated as a function of Li thickness. Figure 5 shows the total neutron yield for each Li thickness. For Li thicknesses between 50 and 90 μm, the total neutron yield was approximately proportional to the Li thickness (correlation coefficient: 1.00).

The residual proton energy after passing through a Li thickness of 50–90 μm ranged from 1.88 to 2.15 meV, and the results were consistent with those of previous report^26^. Hence, the total neutron yield corresponding to the Li thickness (Eq. (3)) can be expressed as

4 $${Y}_{\mathrm{proportional}}^{^{\prime}}\left(t\right)=\left(l\times t+m\right) \quad [\mathrm{mC}^{-1}],$$

where *l* and *m* represent the coefficients, and *t* represents the Li thickness on the Li target. In case of the Li thickness of 50–90 μm, the coefficient of “*l*” represents the increment rate per unit the Li thickness, and the coefficient of “*m*” represents the total neutron yield in the Li thickness of 50 μm.

**Figure 6:**
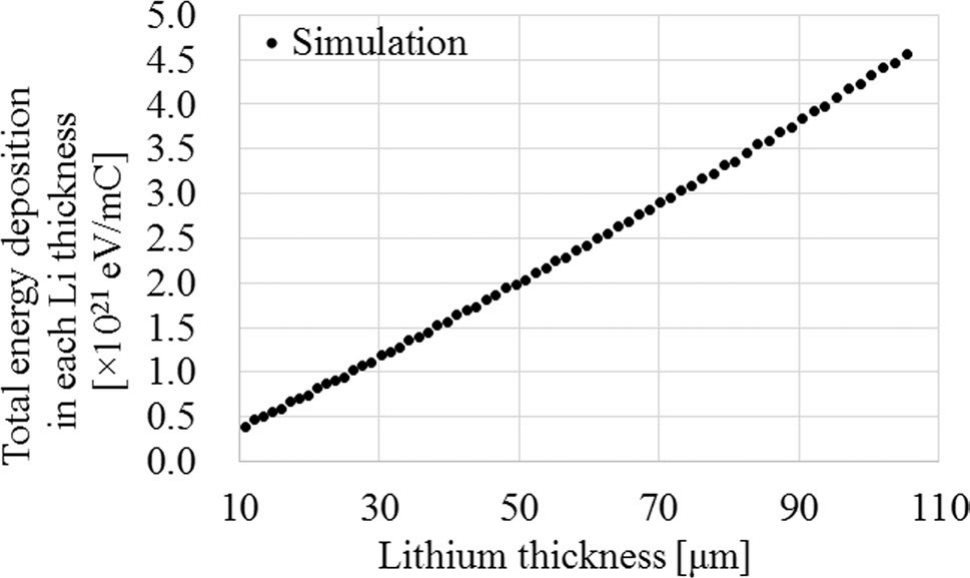
Total energy deposition as a function of Li thickness on the Li target.

Figure 6 shows the total energy deposition after passing through a certain Li thickness.

According to Fig. 6, the total energy depositions were approximately proportional to the Li thickness (Correlation coefficient: 1.00). Additionally, the total energy deposition depends on the number of delivered protons, and the degradation is proportional to the total energy deposition^27^. Therefore, the variation of the Li thickness is represented as a function of the total number of delivered protons, and it can be then represented as follows:

$$\frac{dt}{dp}=-\lambda t$$

5 $$\iff t(p)={t}_{\mathrm{initial}}\times {\mathrm{e}}^{-\lambda p} \left(p=0\to t={t}_{\mathrm{initial}}\right)$$

where *t* denotes the Li thickness on the Li target, *t*_initial_ denotes the Li thickness on the Li target with an initial condition (i.e., no protons delivered to the target sample), *p* denotes the total number of protons delivered to the target sample, and *λ* denotes the coefficient of the degradation that induces the thinning of the Li layer on the Li target. By substituting Eq. (5) into Eq. (4), the total neutron yield becomes a function of the total number of protons delivered to the target sample. Hence, the total neutron yield as a function of the total number of proton charge delivered to the target sample can be expressed by Eq. (6).

6 $${Y}_{\mathrm{proportional}}^{^{\prime}}\left(p\right)=\left(l\times {t}_{\mathrm{initial}}\times {\mathrm{e}}^{-\lambda p}+m\right) \quad [\mathrm{mC}^{-1}]$$

To determine the model for the neutron flux, the unit of the total neutron yield was converted to mA^–1^, and several coefficients in Eq. (6) were simplified as follows:

7 $$F\left(mAh\right)=a\times \mathrm{exp}\left(-b\times mAh\right) +c [{\mathrm{mA}}^{-1}]$$

where *F* represents the neutron flux per unit of proton current based on the total number of protons delivered to the target sample, *mAh* represents the total number of protons delivered to the target sample, and *a*, *b*, and *c* denote the coefficients that reflect the condition of the Li target and the reaction between the Li target and delivered protons.

### Validation of the model for neutron flux using the measured neutron flux

**Figure 7:**
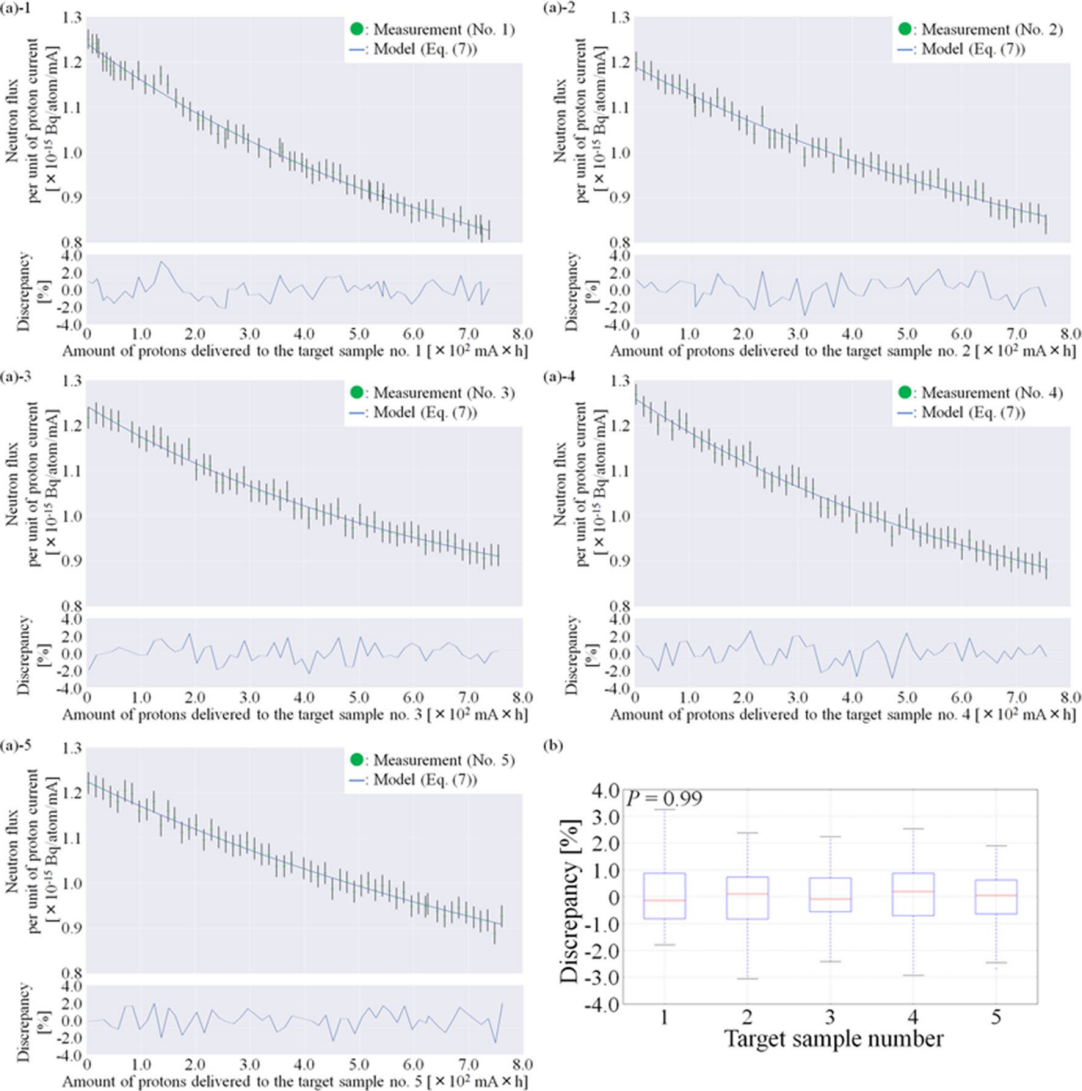
Comparison between the measured and the calculated neutron flux based on the total number of protons delivered to the target sample. (**a**) Comparison in each target sample and (**b**) box plot of the discrepancies in each target sample. The calculated neutron flux was determined by the established model for neutron flux in each target sample, and the saturated radioactivity of ^198^Au was used as a substitute for the neutron flux.

To examine the model for neutron flux, it was applied to the measured neutron flux in each Li target, and those coefficients were then determined in each target sample using the least-square method. Thus, the measured and calculated neutron flux per unit of proton current at each of the total number of delivered protons could be compared. The comparisons between the measured and the calculated neutron flux in each target sample are shown in Fig. 7a, and the discrepancies in each target samples are shown in Fig. 7b.

Based on the comparisons between the measured and calculated neutron flux at each of the total number of delivered protons, the discrepancies in each target sample followed a normal distribution (*p* = 0.60, 0.50, 0.89, 0.89, and 0.29). The mean discrepancy in all cases was less than 0.1%, and the standard deviations (SDs) for each of the five target samples were 1.1, 1.2, 1.0, 1.2, and 1.1%, respectively. Therefore, the neutron flux in each target sample could be represented by the model for each of the total number of protons delivered to the target sample. The acquired model in each target sample is listed in Table 1.

**Table 1:** Model for the neutron flux in each target sample.

Target	Acquired formula	Coefficient		
		*a*	*b*	*c*
1	*F*(*mAh*) = 6.75 × 10^–16^exp(–1.29 × 10^–3^ × *mAh*) + 5.67 × 10^–16^	6.75 × 10^–16^	1.29 × 10^–3^	5.67 × 10^–16^
2	*F*(*mAh*) = 6.08 × 10^–16^exp(–1.05 × 10^–3^ × *mAh*) + 5.82 × 10^–16^	6.08 × 10^–16^	1.05 × 10^–3^	5.82 × 10^–16^
3	*F*(*mAh*) = 4.93 × 10^–16^exp(–1.50 × 10^–3^ × *mAh*) + 7.52 × 10^–16^	4.93 × 10^–16^	1.50 × 10^–3^	7.52 × 10^–16^
4	*F*(*mAh*) = 5.74 × 10^–16^exp(–1.40 × 10^–3^ × *mAh*) + 6.87 × 10^–16^	5.74 × 10^–16^	1.40 × 10^–3^	6.87 × 10^–16^
5	*f*(*mAh*) = 6.19 × 10^–16^exp(–9.44 × 10^–4^ × *mAh*) + 6.07 × 10^–16^	6.19 × 10^–16^	9.44 × 10^–4^	6.07 × 10^–16^

*mAh* is the total number of protons delivered to the target sample. The saturated radioactivity was used as a substitute for the neutron flux.

The discrepancies between the measured and calculated neutron flux at each of the total number of delivered protons were normally distributed among the five target samples (*p* = 0.59). According to the result of the one-way ANOVA test, the discrepancies among the five target samples did not show a statistically significant difference (*p* = 0.99). Therefore, the neutron flux model could be applied to each of the target samples with comparable accuracy.

The neutron flux model derived from the measured neutron flux at the integrated proton charge of up to 60, 100, 200, 300, 400, 500, and 600 mA × h, respectively, and the neutron flux after the amount of delivered protons of 12 mA × h (i.e., 12 mA × 1 h irradiation) was then calculated. It was compared with that calculated via the model derived from the measured neutron flux at the integrated proton charge of up to 750 mA × h. The discrepancies among the five target samples at the integrated proton charge of 60, 100, 200, 300, 400, 500, and 600 mA × h were less than 3.1%, 2.0%, 1.6%, 1.0%, 0.9%, 1.0%, and 1.5%, respectively. Thus, the discrepancies were improved until the integrated proton charge of 300–400 mA × h.

In the cross-validation, the comparisons between the measured and calculated neutron flux, which utilized the model established by the other target samples, in each target sample are found in Supplementary Fig. S1(a), and the discrepancies in each target sample are found in Supplementary Fig. S1(b). Based on the comparisons between the measured and calculated neutron flux, which utilized the model established by the other four target samples, at each of the total number of delivered protons, the discrepancies in the target samples No. 1, 2, 3, and 4 followed a normal distribution (*p* = 0.35, 0.72, 0.16, and 0.80), the mean discrepancies were − 3.8%, − 3.1%, 2.4%, and 1.7%, and the SDs were 3.1%, 1.4%, 1.4%, and 1.3%. In target sample No. 5, the discrepancies did not follow a normal distribution (*p* = 0.02), and the median discrepancy (1st–3rd quartile) was 3.1% (1.9–4.1%). Additionally, a distribution of the discrepancies of the cross-validation was examined in each target sample. In the target sample No. 1–4, the discrepancy was distributed on around its mean value (No. 1: − 3.8%, No. 2: − 3.1%, No. 3: 2.4%, No. 4: 1.7%). On the other hand, in the target sample No. 5, the discrepancies were distributed on around − 0.8% and 3.9%. Values of the measured neutron flux were smaller than those of the calculated neutron flux acquired by the cross-validation model when the total number of protons delivered to the target sample is small, such as less than 140 mA × h. This might be associated with the difference of the degradation in the Li material in each target sample. From Table 1, the reduction of neutron flux along the total number of protons delivered to the target sample No. 5 is smallest among the five target samples. Thus, the discrepancies did not follow normal distribution in the target sample No. 5 although the discrepancies in the target sample No. 1 were larger than those in the target sample No. 5. Furthermore, the discrepancies between the measured and calculated neutron flux, which utilized the neutron flux model established by the other four target samples, at each of the total number of delivered protons, were not normally distributed among the five target samples (*p* <  < 0.001). According to the results of the Kruskal–Wallis test, the discrepancies among the five target samples showed a statistically significant difference (*p* <  < 0.001). Therefore, the coefficients in the neutron flux model has to be determined by results of neutron flux measurements in each target sample rather than those in the other target samples although the same model can be applied.

## Discussion

Previous reports suggest that there are some challenges associated with neutron production in the accelerator-based BNCT system, which employs a solid-state Li target^20,27,28^. One of the primary difficulties is the reduction of the neutron flux per unit of proton current. According to previous reports, the reduction is mainly induced by the degradation of the Li target because a large number of protons are required to be delivered to the target sample to obtain the requisite number of neutrons for BNCT^20,28^. A reduction in the neutron flux per unit of proton current was also observed in this study. Although the reduction of the neutron flux may not be avoidable in the accelerator-based BNCT systems using a solid-state Li target, there still exists the possibility of making further improvements, such as the improvement of the target sample and proton delivery method onto the target sample.

### Clinical implementation of the neutron flux model

According to a previous study, the saturated radioactivity of gold can be a substitute for the neutron flux in the accelerator-based BNCT system with various Li thicknesses even if the neutron capture reaction of the gold depends on the neutron energy^23^. This study investigated that the model based on the measurements of the saturated radioactivity can appropriately represent the neutron flux for each of the total number of protons delivered to the target sample (Fig. 7). Additionally, it is a possibility that other measurement data is substitute for the measured neutron flux, and it can be then applicable to the model. The induced radioactivity of ^7^Be on the Li target reaches 10^11^ Bq at the integrated proton charge of 700 mA × h, and it may be one of the substitutions. The previous study also indicated that a significant reduction of the neutron flux was associated with the destruction of the target sample in the accelerator-based BNCT system^20^, and its reduction was different from the trend of the reduction that was represented by the model in this study. Hence, the model also compensated for the stable operation of the accelerator-based BNCT system. When it is applicable to the clinical, it is important for the clinical application to establish the model easily. The advantage of establishing method for the model is that it can be established with the neutron flux measured at only a single defined distance while some detectors such the measurements at some distance from the target are existed and available. Additionally, it is also the advantages in establishing the model that it requires a smaller amount of neutrons to measure the neutron flux. Because the neutron flux is reduced by the delivered protons to the target sample, the smaller amount of neutrons for the measurement is preferred. When the distance between the target sample and the detector is increased, the neutron flux on the detector is decreased. As a result, when the measurement data at some distance is required for establishing the model, a larger amount of neutrons may be required. Therefore, considering the clinical usage of the model, it is useful to establish the model using the measured neutron flux at only a single defined distance in the accelerator-based BNCT system employing the Li target.

Additionally, the previous study also reported that the degradation of the Li target did not affect the BNCT doses when the required neutron fluence was delivered to the patient^23^. In BNCT, the absorbed doses induced by the reaction of ^10^B(n, α)^7^Li, ^14^N(n, p)^14^C, (n, n′), and ^1^H(n, g)^2^H may depend on the Li thickness. The previous study investigated that the variation of Li thickness did not affect the reaction of ^10^B(n, α)^7^Li, ^14^N(n, p)^14^C, and ^1^H(n, g)^2^H while it was affected to the reaction of (n, nʹ) (i.e., hydrogen dose)^23^. The hydrogen dose differed by as much as 4% due to the variation of Li thickness^23^. However, according to a previous study, the hydrogen dose reached 21% of the total delivered dose in the maximum^40^. Therefore, variation of the Li thickness affects the BNCT dose of less than 1% in the system. A previous study recommended that the uncertainty of the total delivered dose was less than 5% to avoid impacting clinical outcome in radiation therapy with X-ray^41^. Thus, no notable effect induced by the variation of the Li thickness is expected to the clinical effect although the discussion about an overall uncertainty of BNCT needs to be considered. Therefore, the established model is important in the accelerator-based BNCT system employing the solid-state Li target to deliver the required neutrons and BNCT dose to a patient.

The model has the ability to predict by how much the neutron flux is reduced during the treatment using the accelerator-based BNCT system employing the solid-state Li target. In the case of clinical use of the system, a predetermined neutron fluence must be delivered to a patient. Using the model, the predetermined neutron fluence can be calculated as the number of protons delivered to the target sample during the treatment. Thus, the reduction of neutron flux during the treatment can be corrected using the number of protons delivered to the target sample before the treatment, and the predetermined neutron fluence can be delivered to the patient. However, there are some problems in utilizing the model established by the measured neutron flux in each target sample when the reduction of neutron flux during BNCT is predicted by the model before the treatment. It is necessary to assume that the model is established based on the results of the measured neutron flux before each treatment. In particular, we have to carefully discuss the accuracy of the model when the total number of protons delivered to the target sample is small, such as less than 60 mA × h. This is because a small reduction is expected, and the accuracy may not be sufficient as the uncertainty of the measured neutron flux reaches 2.60%. According to the measured neutron flux (Fig. 2), approximately 60 mA × h proton irradiation to the target sample is required to reduce the neutron flux by 2.60%. Therefore, it is necessary to discuss how much of the total number of protons delivered to the target sample is required to determine beginning of the clinical use while considering the prediction accuracy and effects to a patient. On the other hand, according to a previous study, it takes less than 1 h to perform BNCT in a patient using the system^27^. Thus, the reduction of neutron flux is less than 1% during the treatment. We also have a choice to treat a patient without the neutron flux model when the total number of delivered protons is small. Additionally, based on the comparison of the neutron flux model at the integrated proton charge of up to 60, 100, 200, 300, 400, 500, and 600 mA × h, respectively, with that of up to 750 mA × h, the discrepancies were improved until the integrated proton charge of 300–400 mA × h. Although it seems that the accuracy of the model derived from the integrated proton charge of 60 mA × h is sufficient, the required accuracy for the model will be discussed with considering the clinical effect and the overall uncertainty of BNCT in future work. Considering these factors, this study focused only on the validity of the neutron flux model.

As a result of the cross-validation, the reductions in each target sample could not be sufficiently represented by the neutron flux models established by the cross-validation (see Supplementary Fig. S1). On the other hand, it could be represented by the measurement result in each target sample (Fig. 7). These differences might be because the lot number was different for each target sample. A previous study indicated that the reduction rate of neutron flux along the total number of protons delivered to the target sample depended on the proton profile delivered to the Li target, and its rate was considerably higher than those in this study when the proton profile was small^20^. The reduction was examined by tuning the proton profile in the previous study^20^. Although this study did not tune the proton profile, the differences of the reduction of neutron flux along the total number of protons delivered to the target sample were also observed. According to the neutron flux model developed in this study, the coefficients of “*a*,” “*b*,” and “*c*” in its model reflected the Li target condition. According to Table 1, the coefficients of “*a*,” “*b*,” and “*c*” were different among the five target samples, and maximum differences of “*a*,” “*b*,” and “*c*” reached 37%, 59%, and 33%, respectively. Thus, the difference of the reduction rate was also associated with the lot number even if each of the target samples had the geometry and parameters as given in Fig. 1 and the manufacturing process was same. Additionally, if each coefficient in the model can be determined uniquely, the measurement of the neutron flux is not necessary but the integrated proton charge on each target sample has to be measured to control the neutron flux in the system. Thus, the uncertainty in the measurement of the saturated radioactivity can be reduced. However, these results indicated that those coefficients were not determined uniquely. Therefore, the neutron flux model should be established based on the measured neutron flux in each target sample.

### Interpretation of the neutron flux model

In this study, Eq. (3) was used to investigate the relationship between the thickness of the Li target and neutron production. Thus, the main reason for the observed reduction maybe the variation of the Li material in the target. This is consistent with previous studies^20,23^. The collisions between the protons and the Li target introduce a certain amount of thermal load into the Li target, which may cause the thinning of the Li target. As shown in Fig. 4, a Li thickness of 90 μm is required to decelerate 2.50 meV protons to the threshold energy (1.88 meV) of the ^7^Li(p, n)^7^Be reaction^25,29^. In addition, Fig. 5 shows that when the Li thickness on the Li target is reduced, a corresponding reduction in the total neutron yield is observed. The total neutron yield was found to be approximately proportional to the Li thickness when its thickness ranged from 50 to 90 μm. Additionally, a previous study suggested that a Li thickness of more than 20 μm was correlated to the total neutron yield and the saturated radioactivity of the gold standard at the patient position^23^. Thus, in this range, the total neutron yield can be expressed by Eq. (4), instead of Eq. (3). Furthermore, according to a previous report^27^, the degradation correlates to the total energy deposition, and from Fig. 6, the total energy deposition depends on the Li thickness on the Li target. Hence, the Li thickness after a certain amount of proton bombardment can be represented by Eq. (5). Therefore, the magnitude of the reduction of the neutron flux depends on the Li thickness and the number of protons delivered to the target sample, as in Eq. (6). Moreover, according to Fig. 5, the total neutron yield depends on the residual proton energy after passing through a certain Li thickness. According to Fig. 3 and the previous report^26^, the total neutron yield with the Li thickness of 50 μm, in which the residual proton energy was 2.15 meV after passing through the Li target, was comparable to the number of neutrons generated from the Li thickness between 50 and 90 μm. The residual proton energies after passing through the Li thickness of 50 and 90 μm were 2.15 and 1.88 meV, respectively. From Table 1, the acquired coefficient of “*a*” in Eq. (7) was comparable to that of “*c*” for each target sample. Therefore, at residual proton energies between 1.88 and 2.15 meV, the total neutron yield can be expressed using the exponential term in Eq. (7), while the other term can be used to express the neutron generated reaction at residual proton energies above 2.15 meV, even though it is derived as the integration constant in Eq. (7). Hence, if the Li target has only the Li thickness of less than 50 μm, the exponential term may not contribute to the model. In this case, a certain calculation for considering the reduction of neutron flux (i.e., Li thickness) may have to be added into the other term (the coefficient of “*c*” in the model). Moreover, the exponential term may not be needed in the model because the exponential term represented the neutron production over the Li thickness of 50 μm. It may be observed in the system when the integrated proton charge on the Li target is increased (> 750 mA × h). Those data was not measured because the lifetime of each Li target was determined as 700 mA × h in the system. However, it is interesting for the neutron source using the ^7^Li(p, n)^7^Be reaction. Hence, it will be clarified in future work. Additionally, for the incident proton energy of more than 2.5 meV, the model may have to consider neutron generation from ^7^Li(p, n)^7^Be*^29^.

The longitudinal and lateral straggling can be observed when the proton path through any medium. Those straggling can be calculated via Monte Carlo simulations (SRIM-2013). When the proton energy of 2.5 meV is injected into the Li target, the longitudinal and lateral straggling reach 9.7 and 4.5 μm, respectively. In case of the Li thickness of less than approximately 100 μm, the longitudinal straggling affects the neutron production because the Li thickness of 90 μm is required to decelerate 2.50 meV protons to the threshold energy of ^7^Li(p, n)^7^Be reaction and its straggling is 9.7 μm. Additionally, in case of the Li thickness of less than approximately 85 μm, the lateral straggling affects the neutron production because a path length of the incident proton in the Li thickness is extended. However, the model established by the measured neutron flux reflects a mean interaction path length of the incident protons in the Li target rather than the Li thickness. Therefore, the established model can consider those straggling.

### Relationship between the neutron flux model and the thermal load on the target sample

This study investigated whether the model for the neutron flux could represent the reduction of neutron flux in an accelerator-based BNCT system employing a solid-state Li target. Equations (6) and (7) reflect the thermal load delivered to the target sample, and the thermal load then induces the degradation of the Li target. The large thermal load induces various reactions, such as evaporation, chemical reactions, radiolysis, and etc. There was no data given to specify the reason for the reduction of neutron flux in more detail than that due to the thermal load on the Li target. However, the previous study investigated that the reduction rate of neutron flux depended on the proton beam profile on the Li target, and the smaller proton beam profile was associated with the higher reduction rate while the proton current was same^20^. Thus, the reduction rate depended on the thermal load on the Li target, and its reduction was induced by those various reactions related to the large thermal load on the Li target. Therefore, the reduction of neutron flux in the accelerator-based BNCT system with a solid-state Li target may also be improved by increasing the cooling efficiency of the Li target because the thermal load become lower. If the cooling efficiency is improved in the accelerator-based BNCT system, the model for the neutron flux may be utilized although the coefficient (“*b*” in Eq. (7)) is changed.

### Limitations

The limitations of this study are as follows. The proton current was restricted to 12.0 mA, and the cooling efficiency of the Li target was ignored. The previous study investigated that the reduction rate of neutron flux depended on the proton beam profile on the Li target, and the smaller proton beam profile was associated with the higher reduction rate^20^. Those data might be substitute for that in the injected proton current of higher/lower than 12 mA. Therefore, the reduction is associated with the thermal load on the Li target. Thus, we expect that the model can be utilized by changing the coefficients even if a higher proton current is adopted. As a result, the coefficients corresponding to the reduction in Li thickness may be higher. With regard to the cooling efficiency, if considered, the coefficient of “*λ*” in Eq. (5) and “*b*” in Eq. (7) may change because the total energy deposition in the Li target is reduced.

## Conclusions

This study focused on the neutron output characteristics in an accelerator-based BNCT system with a solid-state Li target. The neutron output is reduced by the proton current delivered to the target sample. However, the reduction can be represented by the model established in this study. Hence, in the accelerator-based BNCT system employing the solid-state Li target, there is a possibility that the decrement of the neutron flux during BNCT can be predicted by the model established before each of its treatments although the required accuracy of prediction will be discussed in future work. Additionally, by selecting the adequate coefficients in the model or adequately revising the model, it may be applicable to other BNCT systems employing the Li target, thereby promote research on such system. Therefore, the established model plays an important role in the accelerator-based BNCT system to perform BNCT and understand the neutron output characteristics, and may contribute to improving therapeutic efficacy and safety of BNCT.

## Supplementary Information

Supplementary Information.
