# Supplementary material for: Neutron flux evaluation model provided in the accelerator-based boron neutron capture therapy system employing a solid-state lithium target
Source: Sci Rep. 2021 Apr 13;11:8090. doi: 10.1038/s41598-021-87627-8 (PMC8044165; doi:10.1038/s41598-021-87627-8)
Supplement: Supplementary file 1 — Supplementary Information. [file 41598_2021_87627_MOESM1_ESM.docx]

Manuscript for *Scientific reports*

Article type: Research article

Title:

Neutron flux evaluation model provided in the accelerator-based boron neutron capture therapy system employing a solid-state lithium target

Running title: Neutron flux in a Li target BNCT system

Authors:

Satoshi Nakamura^1, 2*^, PhD., Hiroshi Igaki^2, 3^, MD, PhD, Masashi Ito^4^, Shoji Imamichi^2, 5^, PhD, Tairo Kashihara, MD, PhD, Hiroyuki Okamoto^1, 2^, PhD, Shie Nishioka^1, 2^, PhD, Kotaro Iijima^1^, Takahito Chiba^1, 6^, MS, Hiroki Nakayama^1, 6^, MS, Mihiro Takemori^1, 6^, MS, Yoshihisa Abe^2, 7^, Tomoya Kaneda^3^, MD, PhD, Kana Takahashi^3^, MD, PhD, Koji Inaba^3^, MD, PhD, Kae Okuma, MD, PhD^3^, Naoya Murakami^3^, MD, PhD, Yuko Nakayama^3^, MD, PhD, Mitsuko Masutani^2, 5, 8^, PhD, Teiji Nishio^9^, PhD, and Jun Itami^1, 2, 3^, MD, PhD

Institutional affiliations:

*^1^ Department of Medical Physics, National Cancer Center Hospital, Tsukiji 5-1-1, Chuo-ku, Tokyo, 104-0045, Japan*

*^2^ Division of Research and Development for boron neutron capture therapy, National Cancer Center Exploratory Oncology Research & Clinical Trial Center, Tsukiji 5-1-1, Chuo-ku, Tokyo, 104-0045, Japan*

*^3^ Department of Radiation Oncology, National Cancer Center Hospital, Tsukiji 5-1-1, Chuo-ku, Tokyo, 104-0045, Japan.*

*^4^ Department of Radiology, National Center for Global Health and Medicine, Toyama 1-21-1, Shinjuku-ku, Tokyo, 162-8655, Japan*

*^5^ Division of Cellular Signaling, National Cancer Center Research Institute, Tsukiji 5-1-1, Chuo-ku, Tokyo, Japan*

*^6^ Department of Radiological Science, Graduate School of Human Health Sciences, Higashi-ogu 7-2-10, Arakawa-ku, Tokyo, 116-8551, Japan*

*^7^ Department of Radiological Technology, National Cancer Center Hospital, Tsukiji 5-1-1, Chuo-ku, Tokyo, 104-0045, Japan*

*^8^ Department of Molecular and Genomic Biomedicine, Nagasaki University Graduate School of Biomedical Sciences, Sakamoto 1-7-1, Nagasaki, 852-8588, Japan*

*^9^ Division of Health Science, Graduate School of Medicine, Osaka University, Yamadaoka 1-7, Suita-shi, Osaka, 565-0871, Japan*

Corresponding author’s information:

Name: Satoshi Nakamura, Ph.D.

Address: Tsukiji 5-1-1, Chuo-ku, Tokyo, 104-0045, Japan

TEL: +81(3)3542-2511, FAX: +81(3)3545-3567, E-mail: [satonaka@ncc.go.jp](mailto:satonaka@ncc.go.jp)

Supplementary File:


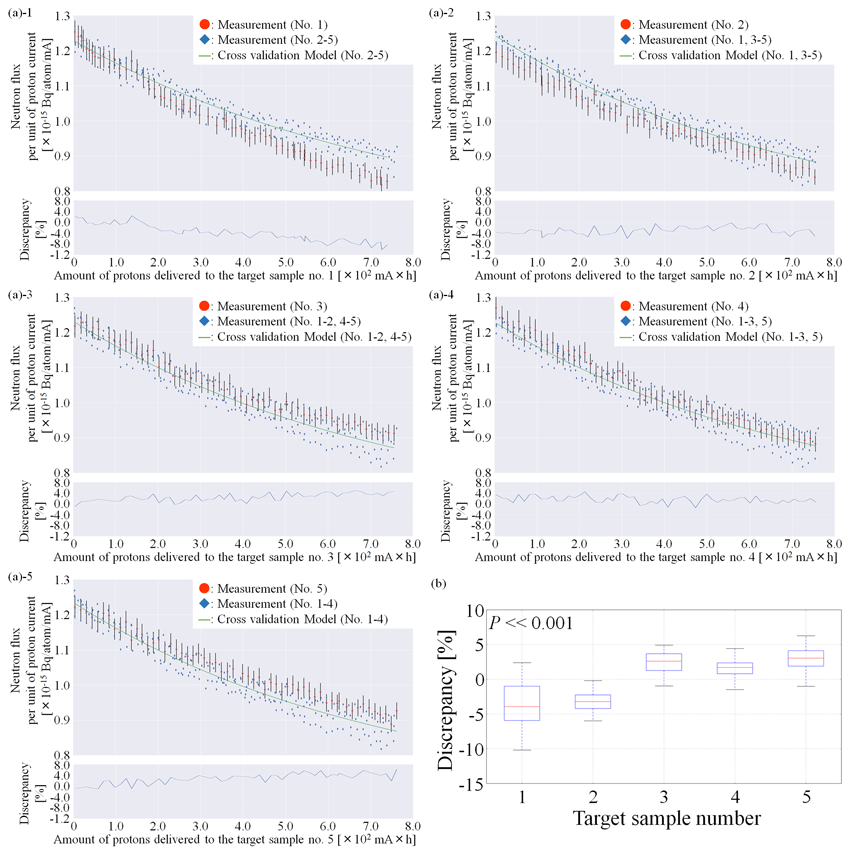


Supplemetary Fig. S1: Comparison between the measured and the calculated neutron flux, which utilized the neutron flux model established by the other target samples, along the total number of protons delivered to the target sample. (a) Comparison in each target sample and (b) box plot of the discrepancies in each target sample. The calculated neutron flux in each target sample was acquired by the neutron flux model established by the other target samples, and the saturated radioactivity of ^198^Au was used a surrogate for the neutron flux.
